# Wanted: studies on mortality estimation methods for humanitarian emergencies, suggestions for future research

**DOI:** 10.1186/1742-7622-4-9

**Published:** 2007-06-01

**Authors:** Working Group for Mortality Estimation in Emergencies

**Affiliations:** 1 Epicentre, 8 rue Saint-Sabin, 75011 Paris, France

## Abstract

Measuring rates and circumstances of population mortality (in particular crude and under-5 year mortality rates) is essential to evidence-based humanitarian relief interventions. Because prospective vital event registration is absent or deteriorates in nearly all crisis-affected populations, retrospective household surveys are often used to estimate and describe patterns of mortality. Originally designed for measuring vaccination coverage, the two-stage cluster survey methodology is frequently employed to measure mortality retrospectively due to limited time and resources during humanitarian emergencies. The method tends to be followed without considering alternatives, and there is a need for expert advice to guide health workers measuring mortality in the field.

In a workshop in France in June 2006, we deliberated the problems inherent in this method when applied to measure outcomes other than vaccine coverage and acute malnutrition (specifically, mortality), and considered recommendations for improvement. Here we describe these recommendations and outline outstanding issues in three main problem areas in emergency mortality assessment discussed during the workshop: sampling, household data collection issues, and cause of death ascertainment. We urge greater research on these issues. As humanitarian emergencies become ever more complex, all agencies should benefit from the most recently tried and tested survey tools.

## Background

Measuring rates and circumstances of population mortality is an essential component of evidence-based humanitarian relief interventions. Crude and under-5 mortality rates are used internationally to benchmark the severity of crises [1] and evaluate the effectiveness of humanitarian assistance. Prospective vital event registration is absent or deteriorates in nearly all crisis-affected populations and as a result, retrospective household surveys are often used to estimate and describe patterns of mortality.

Despite their widespread use and policy implications [2-4], methods behind retrospective mortality surveys have been validated only in specific contexts through comparison with other concurrent surveys or with alternative data sources that happen to be available. Research to optimise mortality survey methods has been scant. Survey estimates have rarely been compared with a proven 'gold standard' mortality rate measurement system. Different types of questionnaires have been proposed, but their performance in various cultural and crisis settings has been insufficiently evaluated. Furthermore, even when the methodology is uncontroversial, reviews of field surveys of nutritional status, immunisation, and HIV-related behaviours have highlighted frequent problems of imprecision and bias due to non-adherence to best practices [5-8].

Current mortality survey approaches essentially feature three methodological components: (i) selection of a representative sample of households in the population, using a given design (usually cluster sampling); (ii) a questionnaire administered to household respondents enquiring about deaths and other demographic indicators during a given recall period; and (iii) an attempt, however crude, to determine the cause of any reported deaths. Analysis consists of computing mortality rates (deaths by person-time of exposure) and their design-adjusted confidence intervals over the recall period, describing reported causes (and often circumstances) of deaths, and, where appropriate, projecting findings to the population and time period they are representative of, so as to estimate overall death tolls.

These surveys are often used to determine intervention strategies and advocate for respect of humanitarian law statutes, and in order to best inform decision-makers the data obtained must be valid. Here we review some of the inherent methodological challenges in the conduct of mortality surveys in humanitarian emergencies, propose best practices, and recommend new research to improve on current methods. Other important issues, such as ways to improve questionnaire design or interview techniques and preparation, are not discussed here. We proceed by tackling the following issues: problems in sampling, household data collection, and cause-of-death ascertainment. The paper represents the output of a June 2006 workshop hosted by Epicentre and funded by Médecins sans Frontières in Veyrier-du-Lac, France.

## Analysis

### 1. Sampling problems in mortality surveys

#### 1.1. Choice of sampling design

##### 1.1.1. Simple or systematic random sampling

Simple random sampling, whereby *n *elements (e.g. households) are randomly selected from a list of the total elements, *N*, is the gold standard of representative sampling, but can only be performed if a list of all individual households with a unique identifier is available. When the area to be covered by the survey is large or the population is very unstable, as in a recently formed displacement camp, creating such a list may be extremely costly and time-consuming, thus defying one of the main purposes of surveys, which is to furnish rapid estimates. Systematic sampling, whereby each *n*th household is sampled, is the second best choice: it requires only knowledge of the total number of households, but is mainly feasible if households are laid out along clearly defined streets or in a clear pattern.

Simple and systematic random sampling designs are explained fully elsewhere [9] and, as their application for mortality estimation does not present specific challenges, they are not explored further here. Although they may be practicable only in some situations, investigators should always consider these two options first, especially if a sampling frame of households can readily be constructed. Analysis of a simple or systematic random sample is straightforward, robust and amenable to various statistical analyses, including post-stratification. These sampling designs also facilitate adequate sample size calculation, as they do not require any assumption about clustering of deaths (see below).

##### 1.1.2. Cluster sampling

Cluster sampling should be used only if simple or systematic random sampling techniques are prohibited by cost, time or logistical constraints. In multi-stage cluster sampling, the entire sampling universe is firstly divided into well-defined areas such as districts or villages. The required numbers of primary sampling units (PSUs or clusters) are then selected from these areas using probability proportional to size (PPS), and occasionally using spatial (random co-ordinate) sampling. Most survey statisticians accept PPS cluster allocation as being a valid approach, although with some caveats, such as the accuracy of the population size data from which the sampling occurs. The final stage of sampling consists of selecting a given number of basic sampling units (in mortality surveys, these are usually households) within a cluster. We focus here on one approach for household selection that has led to much discussion.

In 1978, the World Health Organization's Expanded Programme on Immunization (WHO EPI) adopted the "30 by 7" two-stage cluster sample survey design for rapidly estimating vaccine coverage (VC) in children aged 12 to 23 months [10]. Manuals and step-by-step guidelines [11,12] were subsequently developed and the method has since become widely used [13-19]. Briefly, the WHO EPI "random walk" approach for household selection involves choosing a random direction from the centre of the community and then selection of one household at random along an imaginary line connecting the centre to the periphery. Subsequent households are then selected by proximity (the next nearest household) or visiting every *N*th closest household until the sample size for the cluster is achieved [12].

The EPI method has since been adapted for use in measuring nutritional status [20,21], retrospective mortality [22,23] and other variables [7,24,25]. For non-VC surveys, a sample size of 900 tends to be used (30 clusters of 30 households or children, depending on the variable being measured). Despite recommendations to improve and adapt second stage sampling for non-EPI purposes [17,26,27], over time it has become common practice to apply this survey method without questioning its appropriateness for the outcome being measured.

The main problem with the random walk approach followed by proximity sampling is that the probability of a household being selected is unknown, since the total number of households in the community is unknown. Preferred methods entail selecting a fixed number of households in a cluster so that the probability of selection can be calculated. Segmentation is one such method. The defined area to which the cluster has been allocated is further divided into segments of approximately equal size, at a level at which a complete listing of households in the segment can be made. One segment is then selected randomly [17], and all households within it may then be selected or, alternatively, the desired number of households to make up a cluster may be selected using simple or systematic sampling. However, segmentation is not always possible due to time, security and human resource constraints.

Other methods exist for selection of the first household in the cluster. For example, for sites with an existing map, a "sampling grid" can be superimposed and co-ordinates selected at random [28]. Similarly, GPS units can be used to establish the boundary of the cluster area, and select a random coordinate within it [29,30]. For either of these methods, we recommend selecting subsequent households in the cluster by a different proximity rule – e.g. not next nearest, but every third or fifth household. This would result in greater geographical spread, thus decreasing clustering (see below) and favouring randomness.

#### 1.2. Clustering of risk of death

##### 1.2.1. Why clustering occurs

Risk of death may be clustered, a phenomenon that is probably more pronounced in emergencies; particular villages may suffer disproportionate violent attacks, be different in terms of access to healthcare, or differ in certain epidemic-prone diseases like measles, which may spread from index cases to neighbours and close contacts [31,32]. Thus, there is an increased probability that individuals within clusters will resemble each other (i.e. share a high or low risk of mortality), and differ considerably from individuals in other clusters. This probability increases as the number of clusters selected decreases, and as the sample size per cluster increases. High intra-cluster homogeneity decreases precision, since it results in larger standard errors and correspondingly wider confidence intervals.

##### 1.2.2. How to report on clustering: design effect (Deff) and intra-cluster coefficient (ICC)

**Figure 1:**
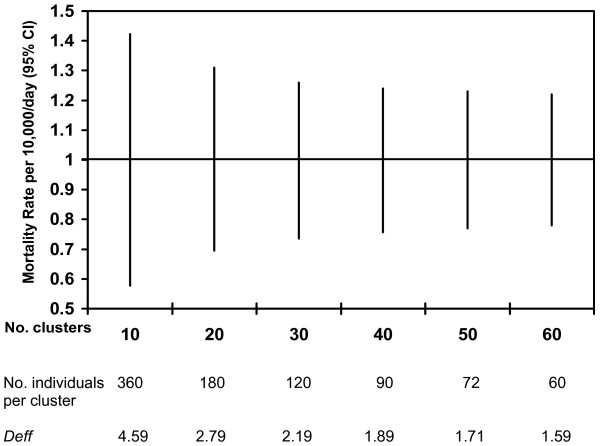
**The effect of the number of clusters and number of individuals per cluster on the 95% CI for a mortality rate of 1/10 000/day (*N *= 3600 individuals). **Note: The ICC can be used to calculate *Deff *for different numbers of individuals per cluster (*m*) using the following formula: ICC = (*Deff *-1)/(*m *- 1).

There are two related measures of clustering within a cluster sample: the ICC and *Deff*. The ICC indicates the likelihood of individuals within the same cluster sharing the same outcome (an ICC of 1 means total clustering, and an ICC of zero means no clustering). The ICC depends on the variance within and between clusters, the actual frequency of the outcome being measured, the cluster size [33], and the number of clusters. Derived from the ICC and the cluster size, *Deff *has been defined as the ratio of the variance taking clustering into account to the variance assuming simple random sampling [34]. The *Deff *has been used more often than the ICC in mortality surveys since it provides an inflation factor for both calculating an adequate sample size before the survey (based on the expected *Deff*), and adjusting confidence intervals after the survey (based on the observed *Deff)*. Figure 1 shows the variation in the 95% confidence interval and *Deff *for different numbers of clusters and varying numbers of individuals per cluster.

There is little published research from mortality studies on observed *Deff *and ICC to provide accessible guidance for non-statisticians in the context of humanitarian emergencies [35]. We recommend that, before any cluster survey, investigators perform a literature search for past reports providing *Deff *or ICC within the population to be studied, so as to inform sample size calculations.  Henceforth, both statistics should be calculated after each survey, and presented in survey reports or publications so as to improve future survey designs.

##### 1.2.3. Adjusting for clustering in analysis

Every stage in sampling (including any sub-segmentation required to home in on a sufficiently small enumeration area) can introduce a further level of clustering; e.g. clustering of deaths within the district, within villages in the district and within neighbourhoods of the village. Additional clustering may even occur within the households themselves; for example, a household in which a mother has died during childbirth is also more likely to experience infant deaths. Most data analyses only take into account variance at the PSU level (the variance accounting for differences between clusters), and ignore the household level. Although it is generally thought that once the variance at the cluster level is accounted for the variance at the household level is minimal, this has been poorly studied. We encourage investigators to assess this assumption in studies of mortality during humanitarian emergencies.

#### 1.3. Stratification

There are a number of reasons to stratify the population under study, including the desire to improve the precision of estimates, assuring subgroup representation in the final sample, and the need to calculate stratum-specific estimates.

In explicit stratification population elements are separated into non-overlapping groups (strata), and sampling is performed within each stratum. This can serve three main purposes. Firstly, it can ensure that each stratum receives a proportionate share of the sample and improves precision by favouring across-strata heterogeneity. This is achieved by classifying the sampling frame into strata according to a variable known to be associated with mortality, and assigning a portion of the sample to each stratum proportionate to its population size. Secondly, it can allow for sub-group analyses by ensuring that enough of the sample is drawn from a given stratum (for example, an ethnic minority). This is done by sampling unequally in certain strata. Thirdly, it can obtain an independent estimate for each stratum (e.g. a sub-region) by drawing a sufficient sample from each. The data can then be pooled into one estimate for the entire sampling universe. In the latter two cases, weighting is needed in order to obtain an arithmetically correct pooled estimate. This causes some loss in precision.

Implicit stratification can also be used to increase precision, and is a preferable alternative to explicit stratification when a continuous variable is known or thought to be associated with the outcome [36]. With implicit stratification the sampling frame of PSUs is ranked according to this continuous stratification variable. The data are analysed after applying Kish's method of paired clusters [34] to define the 'implicit' strata. By pairing the clusters the maximum number of strata of minimum size is created. Each cluster in the pair will therefore be very similar with regard to the variable selected. There will, however, be high between-pair heterogeneity. The more homogeneity exists *within *strata, and the more differences exist *between *strata, the greater the precision benefit of *any *stratification approach. Implicit stratification, however, maximises this benefit, since it yields the highest possible ratio of inter-strata to intra-strata variance, assuming, of course, that the chosen stratification variable is indeed associated with the outcome of interest. The opportunity to perform implicit stratification in cluster sampling occurs before PPS cluster allocation. The procedure below illustrates how to do this.

1. Order the defined areas (e.g. refugee camps) in the sampling frame for PPS cluster allocation according to your hypothesis (e.g. date of arrival if this is thought to be related to better living conditions and thus mortality risk), and perform PPS cluster allocation as usual.

2. At the analysis stage, retain the order of selected clusters as it was in the implicitly stratified sampling frame, and combine them into pairs, working down the list. For example, the first stratum will consist of the first and second clusters, the second stratum of the third and fourth clusters, etc. If there is an uneven number of clusters, the last three clusters are placed in triplet.

3. Treat each pair as if they were explicit strata (there will be half as many explicit strata as there are clusters) and assign to each pair a unique stratum identification number.

4. Perform stratified analysis.

Most software statistics packages provide for analysis by stratum. This functionality is generally used when explicit stratification is performed, whereby strata are explicitly defined prior to sampling and the information regarding the stratum to which each survey subject belongs is already contained in the database. If using implicit stratification on a continuous variable, such as distance or average income, a stratum variable needs to be defined before selection for analysis, and each cluster pair becomes a stratum.

In the example shown in Table 1 we demonstrate simultaneous explicit and implicit stratification to improve estimate precision. Instead of being listed alphabetically or randomly, refugee camps to be assessed as part of a 60-cluster region-wide survey are instead explicitly stratified according to whether they received two, one or no general food distributions during the recall period. This categorical variable is hypothesised to be a correlate of malnutrition, and thus mortality. Three explicit strata (camps with no distributions, camps with one distribution, and camps with two distributions) are created, and a fraction of the total required clusters is allocated to each stratum by PPS. However, it is also hypothesised that the date of establishment of camps is correlated with mortality, since older camps may have better living conditions. Within each explicit distribution stratum, camps are ranked from oldest to most recent. Clusters are then allocated by PPS to each camp, and finally are coupled into implicit stratification pairs, and the odd triplet.

**Table 1:** Example of Implicit Stratification

**Sampling frame of 14 hypothetical refugee camps in the sampling universe (date of establishment)**	**Population**	**Clusters allocated by PPS (total *n *= 60)**	**Implicit stratification pairs for analysis (n=30)**
**Explicit stratum 1: No food distributions during recall period**	**42 000**	**27**	
Camp Romeo (May 2003)	12 000	8 (c1†-c8)	P1.1‡ (c1,c2), P1.2 (c3,c4), P1.3 (c5,c6), P1.4 (c7,c8)
Camp Delta (July 2003)	8000	5 (c9-c13)	P1.5 (c9,c10), P1.6 (c11,c12)
Camp Tango (January 2004)	3000	2 (c14-c15)	P1.7 (c13,c14)
Camp Whiskey (May 2004)	6000	4 (c16-c19)	P1.8 (c15,c16), P1.9 (c17,c18)
Camp Alpha (November 2004)	4000	3 (c20-c22)	P1.10 (c19,c20), P1.11 (c21,c22)
Camp Charlie (February 2005)	9000	5 (c23-c27)	P1.12 (c23,c24), P1.13 (c25,c26,c27)

**Explicit stratum 2: One food distribution during recall period**	**27 000**	**17**	
Camp November (March 2003)	2000	1 (c28)	P2.1 (c28,c29)
Camp India (April 2003)	8000	5 (c29-c33)	P2.2 (c30, c31), P2.3 (c32,c33)
Camp Victor (August 2003)	7000	4 (c34-37)	P2.4 (c34,c35), P2.5 (c36,c37)
Camp Oscar (June 2004)	10 000	7 (c38-c44)	P2.6 (c38,c39), P2.7 (c40,c41), P2.8 (c42,c43,c44)

**Explicit stratum 3: Two food distributions during recall period**	**26 000**	**16**	
Camp Bravo (April 2003)	6000	4 (c45-c48)	P3.1 (c45,c46), P3.2 (c47,c48)
Camp Sierra (November 2003)	1000	1 (c49)	P3.3 (c49,c50)
Camp Foxtrot (March 2004)	3000	2 (c50-c51)	P3.4 (c51, c52)
Camp Uniform (December 2004)	16 000	9 (c52-c60)	P3.5 (c53,c54), P3.6 (c55,c56), P3.7 (c57,c58), P3.8(c59,c60)

† c1 = cluster 1, c2 = cluster 2, etc. ‡P1.1 = explicit stratum 1, implicit pair 1, etc.

#### 1.4. Dealing with absenteeism and refusals

##### 1.4.1. Minimising non-response

In any survey there will be some non-responders, due either to absenteeism or refusal to participate. The greater the proportion of non-responders, the higher the risk of selection bias, and the harder it becomes to interpret findings, since in most circumstances one cannot safely assume that non-responders will experience the same mortality risk as those who enter the sample. Investigators should thus attempt to minimise absenteeism and refusals. Surveys should be performed when people will be near their homes, and there should be a call-back procedure in place to deal with absenteeism; for example, survey investigators could return three times to check on absentee households. One way to pre-empt non-response is to inform community leaders and perform community sensitisation about the timing and objective of the survey, before data collection starts. However, this will result in many people staying at home in expectation of the survey, not realising that only a few households will be sampled: the benefits of this strategy need to be weighed against the harms of entire communities missing out on valuable work time.

##### 1.4.2. Inflation versus replacement

Even the best surveys will have a number of non-responders. It may be better, therefore, to assume a certain proportion of absentees or refusals and to 'inflate' the survey sample size in advance accordingly, than to 'replace' non-responding households at the end of a cluster. For example, if the desired sample size is 900 (e.g. 30 clusters of 30 households) and we expect non-response from 10% of households, then the total sample size could be increased to 1000, or by about 4 households per cluster. 30 clusters of 34 households, or 1020 households, should give a final sample of 918 (i.e. 1020 - (0.1 × 1020)), slightly higher than that required for a sample size of 900 (in a 30 × 30 cluster survey). This technique is known as 'inflation'.

Points in favour of inflation are firstly that all households in a cluster will have an equal probability of being selected. Replacement violates this condition, which could become a concern if the enumeration area population is small relative to the intended cluster size. Secondly, the temptation of convenience sampling (i.e. going to households that seem available) may be reduced. Inflation does, however, have two problems. Firstly, there is a risk of not achieving the desired sample size if there are more non-responses than expected. Secondly, there may be a need to use weighting in the analysis to adjust for different cluster sizes occurring as a result of differing patterns of non-response. If non-response is associated with mortality, both replacement and inflation could result in selection bias because non-responders will not be represented in the sample. Inflation may be theoretically less biased than replacement (although there are no data to support this) since it does not result in over-sampling of non-responders. However, it could be argued that by giving greater weight to smaller clusters (i.e. those with high levels of non-response), these smaller clusters will be over-represented; this introduces a similar bias to that of replacement. In practice, the decision to perform weighting after inflation needs to take into account the reasons for non-response to avoid selection bias as a result of over-representation of non-responders. Accurate documentation of the reasons for each instance of non-response should be standard practice in every survey; this information will be key in interpreting findings, assessing the possibility of selection bias, considering the pros and cons of weighting, and could be important in performing sensitivity analyses which take likely mortality levels among non-responders into account.

#### 1.5. Sample size calculation

Sample size calculations should be conducted for each survey. Calculations of sample size should be based on the estimated proportion, the desired confidence level, the anticipated level of non-response and an estimate of either the ICC or *Deff*. This information can then be used to determine the number of households to select, in addition to an estimated proportion of the target group in the population (e.g. children < 5 years of age) and the average household size (if known). Sample size rationale and calculations should be reported when communicating survey results.

For a given number of clusters, increasing the number of households sampled per cluster will improve precision, although this may be counteracted by an increase in *Deff*. An alternative approach for improving precision is to increase the number of clusters, as demonstrated in a recent review of earlier cluster surveys [35]. However, the extra resources needed for this approach (time, more survey teams and so on) should be considered when making the decision between increasing cluster size and selecting more clusters.

### 2. Household data collection issues

#### 2.1. Ascertainment of events and person-time denominator

The number of people living in all surveyed households and the amount of time each of them spent in the sampling universe during the recall period comprise the basic denominator for computing mortality rates. Different methods of eliciting this information have been applied, and are reviewed below as well as by Woodruff [37]. The recall period is discussed in further detail in the World Food Programme (WFP)'s latest manual [38].

##### 2.1.1. Past household census method

In the past household census method all household members present at the beginning of the recall period are listed. The method includes questions about those who have been born since the start of the recall period and those who have out-migrated during the recall period. In-migrants are not usually included [39]. The denominator is calculated as the total number in the household at the start of the recall period, plus half of the newborns, minus half of the deaths and out-migrants, multiplied by the recall period.

##### 2.1.2. Current household census method

In the current household census method, investigators list all household members at the end of the recall period, as well as all births and deaths during the recall period. In-migrants are included in this approach, but out-migrants who are not a part of the household at the end of the recall period are missed. The denominator is calculated as the total number in the household on the day of the survey, minus half of the births, plus half of the deaths, multiplied by the recall period.

##### 2.1.3. Hybrid census method

In both the past and current census methods some exposure time is unaccounted for in the denominator. The hybrid method is a combination of these methods attempting to account for both in- and out-migration. It is described in the Standardised Monitoring and Assessment of Relief and Transitions (SMART) and WFP manuals [39,38].

##### 2.1.4. Modified prior birth history method

A modification to the prior birth history method has been put forward as an alternative tool for estimation of under-5 mortality [40]. Mothers are asked about all the children born in the last five years, and their fate during the recall period. Here the denominator can be calculated as the total number in the cohort of children under five who are alive at the start of the recall period, minus half of the deaths plus half of the births, multiplied by the recall period.

A disadvantage of this method is that orphans are missed, and mortality may thereby be underestimated. Underestimation may also be a problem in situations of very high maternal and child mortality [41,42]. An advantage, however, is that both under 5 mortality during the recall period and that over a 5-year period can be computed. This potentially provides a local pre-crisis baseline.

Generally, we recommend recording individual rather than aggregate data. Although it may appear more time-consuming, listing each individual by sex, age, and date of death or in-/out-migration (as appropriate), is less complex for field teams and facilitates analysis.

#### 2.2. Analytical issues

##### 2.2.1. Calculation of person-time at risk

If the approximate dates can reliably be established, each individual's person-time may be computed. This provides a more accurate denominator, especially when populations vary non-linearly during the recall period [43]. The related indicator of mortality would then be a real rate showing death occurrence per unit of person-time for a specific population size (e.g. number of deaths per 10 000 person-days). Whilst most populations are more or less static cohorts without much in- or out-migration, others undergo massive fragmentation and displacement during the recall period – for example, Hutu refugees who fled across Zaïre in 1994–1997, and ethnic Karen communities who either remained in Burma or fled to Thailand [44,45].

The benefit of having a more accurate estimate by measuring person-time should be weighed against the difficulties involved if the cohort is dynamic. This could involve a more complex questionnaire; for example, when attempting to establish dates for births, deaths, and in- and out-migrations using a local events calendar. A local events calendar is needed if accurate dates are either unknown or cannot be approximated by household respondents.

##### 2.2.2. Age standardisation and presentation of age pyramids

Age-standardised rates are more informative than crude mortality rates, and can be computed using the WHO standard [46]. Whether or not mortality rates are age-standardised, it is important to illustrate the sampled population's age structure using age pyramids. A sex or age group with a clear deficit could indicate recent high mortality, high out-migration, over-representation of other age-groups and/or sex, high mortality several years before the survey, or changes in birth rate. Age pyramids are best constructed using 5-year age-groups [23]. Those under 5 can be shown on a separate age-pyramid by one-year categories to highlight recent trends among newborns.

### 3. Cause of death investigation

#### 3.1. Current approaches

There is currently no standard approach to ascertaining causes of death in emergency mortality surveys. Generally, investigators ask decedents' next-of-kin about the most likely cause of death and record the answer openly or assign it to a pre-defined list of diseases or signs and symptoms, which are sometimes based on local disease terms or informal qualitative investigation. The validity of such household reports is unknown. Recognising this, the SMART protocol recommends classifying causes of death more conservatively, into "violent" and "other" [39]. The verbal autopsy (VA) literature shows that the sensitivity and specificity of the tool for broad categories are much higher than for the more detailed level [47]. However, distinguishing some of the chronic infectious diseases (e.g. AIDS, TB) from other diseases of non-infectious origin (e.g. cancer) still needs a certain level of detail.

#### 3.2.Verbal autopsy

The VA methodology has been used for demographic surveillance and in research settings to estimate cause-of-death (COD) structures for decades. The components of the tool and its application are discussed in detail in other papers [47-51]. Experience with VA in emergencies is scarce, and there are insufficient data to inform about its usefulness in natural or man-made catastrophes. The main limitations of VA in emergencies are its length and complexity. It takes a minimum of 30 minutes to interview a family reporting a death; too long for surveys in emergencies or insecure environments. Additionally, it is a cost- and time-intensive process to derive the COD from the questionnaires because medical doctors or medically trained personnel need to review each form in order to assign diagnoses.

The VA can be a painful process for bereaved relatives. For psychological and ethical reasons, it may not be possible to perform the interview in a few minutes, even if the tool would permit this [48]. In crisis situations, families often experience sudden, unexpected and very recent losses; these require interviewers trained in bereavement techniques and in psychological counselling. Well-trained lay interviewers could return to the household in which a death was noted during the survey; a similar procedure is currently used at many demographic surveillance sites in low-income countries [49]. Another possibility is to set up a focus group at the survey site, using a VA-type process to elucidate the major causes of death in advance of the survey. In some countries (e.g. Iraq) death certificates are available [3]; these aid greatly in identifying causes of death.

## Conclusion

Mortality surveys have an increasing impact on the allocation of humanitarian relief and on shaping international public opinion and government response to major humanitarian crises. Recent years have seen more region-wide and complex survey efforts [2-4,52,53], sometimes resulting in controversial and disputed estimates.

We examined several methodological problems and outstanding issues with retrospective mortality estimation in humanitarian crises. Whilst current state-of-the-art tools can be improved based on available methods, research is needed to further validate existing methods and to explore possible alternatives. For example, the two-stage cluster sample survey methodology should be compared to other methods for measuring mortality. Results from a non-segmented cluster survey design could be compared with those from an exhaustive survey or complete death-reporting system in both high and low mortality settings, with a capture-recapture approach used for verification.

Establishing the minimum number of clusters needed to provide statistically robust estimates for different mortality rates in the field even in conditions of high clustering would be an important step forward. The random walk method (as well as alternative final stage sampling methods such as use of a sampling grid and random GPS point selection) needs to be compared with the gold standard of listing all basic sampling units and then selecting the required number using simple random sampling. Other alternative methods such as the exhaustive case-finding approach [54] should also be fully investigated. Studies are needed which will compare, using the same survey method on the same population, estimates of mortality obtained using past, current and hybrid household census methods with prior birth history methods.

Finally, the current method needs to be compared with the VA by using one method and then following up with the other, in order to determine which is more accurate. The accuracy of determination of COD should be compared using direct questions versus open-ended questions. A simple algorithm (using broad categories) also needs to be validated, by comparing its use against the detailed VA.

Given the potentially huge public health impact of surveys in complex humanitarian emergencies, we call for increased funding and inter-disciplinary, multi-centric collaboration to tackle a much overlooked research agenda.

## Competing interests

The author(s) declare that they have no competing interests.

## Authors' contributions

The idea for this paper came from a June 2006 workshop involving all members of the group (see appendix 1). Angela MC Rose, Rebecca Freeman Grais and Francesco Checchi wrote the paper based on presentations and discussions during the workshop. All members of the group discussed and offered comments on the draft and approved the final manuscript.

## Appendix 1: Contributors

The members of the Working Group for Mortality Estimation in Emergencies are:

Vincent Brown^1^, Francesco Checchi^2^, Evelyn Depoortere^1^, Rebecca Freeman Grais^1^, P. Gregg Greenough^3^, Colleen Hardy^4^, Alain Moren^5^, Leah Richardson^6^, Angela M. C. Rose^1^, Nadia Soleman^7^, Paul B. Spiegel^8^, Kevin M. Sullivan^9^, Mercedes Tatay^10^, Bradley A. Woodruff^11^

^1 ^Epicentre, 8 rue Saint-Sabin, 75011 Paris, France

^2 ^Department of Infectious and Tropical Diseases, London School of Hygiene and Tropical Medicine, Keppel Street, London WC1E 7HT, UK

^3 ^Harvard Humanitarian Initiative, Harvard University, 14 Story Street, Cambridge, MA 02138, USA

^4 ^International Rescue Committee, 122 East 42nd Street, New York, New York 10168-1289, USA

^5 ^Epiconcept, 47 rue de Charenton, 75012 Paris, France

^6 ^United Nations World Food Programme, Via C.G. Viola 68, Parco dei Medici, 00148 Rome, Italy

^7 ^World Health Organization, 20 avenue Appia, 1211 Geneva 27, Switzerland

^8 ^United Nations High Commissioner for Refugees, Case Postale 2500, CH-1211 Geneva 2 Dépôt, Switzerland

^9 ^Rollins School of Public Health, Emory University, Grace Crum Rollins Building, 1518 Clifton Road, Atlanta, Georgia 30322, USA

^10 ^Médecins Sans Frontières, French Section, 8 rue Saint-Sabin, 75011 Paris, France

^11 ^Maternal and Child Nutrition Branch, National Center for Chronic Disease Prevention and Health Promotion, Centers for Disease Control and Prevention, 1600 Clifton Rd, Atlanta, Georgia 30333, USA

## References

[B1] Salama P, Spiegel P, Talley L, Waldman R (2004). Lessons learned from complex emergencies over past decade. Lancet.

[B2] Burnham G, Lafta R, Doocy S, Roberts L (2006). Mortality after the 2003 invasion of Iraq: a cross-sectional cluster sample survey. Lancet.

[B3] Roberts L, Lafta R, Garfield R, Khudhairi J, Burnham G (2004). Mortality before and after the 2003 invasion of Iraq: cluster sample survey. Lancet.

[B4] Coghlan B, Brennan RJ, Ngoy P, Dofara D, Otto B, Clements M, Stewart T (2006). Mortality in the Democratic Republic of Congo: a nationwide survey. Lancet.

[B5] Boss LP, Toole MJ, Yip R (1994). Assessments of mortality, morbidity, and nutritional status in Somalia during the 1991–1992 famine. Recommendations for standardization of methods. JAMA.

[B6] Garfield R (2000). Studies on young child malnutrition in Iraq: problems and insights, 1990–1999. Nutr Rev.

[B7] Spiegel PB, Le P (2006). HIV behavioural surveillance surveys in conflict and post-conflict situations: A call for improvement. Global Public Health.

[B8] Spiegel PB, Salama P, Maloney S, van der Veen A (2004). Quality of malnutrition assessment surveys conducted during famine in Ethiopia. JAMA.

[B9] Levy PS, Lemeshow S (1999). Sampling of Populations.

[B10] Serfling RE, Sherman IL (1965). Attribute Sampling Methods for Local Health Departments.

[B11] Hoshaw-Woodard S Description and comparison of the methods of cluster sampling and lot quality assurance sampling to assess immunization coverage.

[B12] Expanded Programme on Immunisation: The EPI coverage survey: training for middle-level managers.

[B13] Henderson RH, Sundaresan T (1982). Cluster sampling to assess immunization coverage: a review of experience with a simplified sampling method. Bull World Health Organ.

[B14] Salmaso S, Rota MC, Ciofi Degli Atti ML, Tozzi AE, Kreidl P (1999). Infant immunization coverage in Italy: estimates by simultaneous EPI cluster surveys of regions. ICONA Study Group. Bull World Health Organ.

[B15] Viviani S, Binkin N, Carrieri P, Greco D, Salamina G, Salmaso S, Tozzi AE, Niccolini A, D'Argenio P, Maestro A, Carafo L, Lo Monaco R, Sodano L, Pandolfi P, Filipetti F, Manfredi Selvaggi T (1995). Vaccinal coverage for measles and pertussis: a study in 7 regions of Italy [Italian]. Ann Ig.

[B16] Singh J, Jain DC, Sharma RS, Verghese T (1996). Further observations on comparison of immunization coverage by lot quality assurance sampling and 30 cluster sampling. Southeast Asian J Trop Med Public Health.

[B17] Thórdarson   TT, Haraldsson A, Jonsson H, Chola RG, Gunnlaugsson G (2005). Immunization coverage in the Monkey Bay Head zone Malawi [Icelandic]. Laeknabladid.

[B18] Milligan P, Njie A, Bennett S (2004). Comparison of two cluster sampling methods for health surveys in developing countries. Int J Epidemiol.

[B19] Vellinga A, Depoorter AM, Van Damme P (2002). Vaccination coverage estimates by EPI cluster sampling survey of children (18–24 months) in Flanders, Belgium. Acta Paediatr.

[B20] Bennett S, Radalowicz A, Vella V, Tomkins A (1994). A computer simulation of household sampling schemes for health surveys in developing countries. Int J Epidemiol.

[B21] Binkin N, Sullivan K, Staehling N, Nieburg P (1995). Rapid nutrition surveys: how many clusters are enough?. Disasters.

[B22] Alemu W (1993). Neonatal tetanus mortality survey, north and south Omo administrative regions, Ethiopia. Ethiop Med J.

[B23] Rose AM, Grais RF, Coulombier D, Ritter H (2006). A comparison of cluster and systematic sampling methods for measuring crude mortality. Bull World Health Organ.

[B24] Ferrinho P, Valli A, Groeneveld T, Buch E, Coetzee D (1992). The effects of cluster sampling in an African urban setting. Cent Afr J Med.

[B25] Pandav CS, Arora NK, Krishnan A, Sankar R, Pandav S, Karmarkar MG (2000). Validation of spot-testing kits to determine iodine content in salt. Bull World Health Organ.

[B26] Brogan D, Flagg EW, Deming M, Waldman R (1994). Increasing the accuracy of the Expanded Programme on Immunization's cluster survey design. Ann Epidemiol.

[B27] Turner AG, Magnani RJ, Shuaib M (1996). A not quite as quick but much cleaner alternative to the Expanded Programme on Immunization (EPI) cluster survey design. Int J Epidemiol.

[B28] Grais RF, Rose AMC, Guthmann JP (2007). Don't spin the pen: two alternative methods for second stage sampling in cluster surveys in urban zones. Emerg Themes Epidemiol.

[B29] Roberts L, Despines M (1999). Mortality in the Democratic Republic of the Congo. Lancet.

[B30] Grandesso F, Sanderson F, Kruijt J, Koene T, Brown V (2005). Mortality and malnutrition among populations living in South Darfur, Sudan: results of 3 surveys, September 2004. JAMA.

[B31] Ronsmans C, Etard JF, Walraven G, Hoj L, Dumont A, de Bernis L, Kodio B (2003). Maternal mortality and access to obstetric services in West Africa. Trop Med Int Health.

[B32] Santos SM, Barcellos C, Carvalho MS, Flores R (2001). Spatial clusters detection of violent deaths in Porto Alegre, Rio Grande do Sul, Brazil, 1996 [Portuguese]. Cad Saude Publica.

[B33] Katz J, Zeger SL (1994). Estimation of design effects in cluster surveys. Ann Epidemiol.

[B34] Kish L (1965). Survey Sampling.

[B35] Kaiser R, Woodruff BA, Bilukha O, Spiegel PB, Salama P (2006). Using design effects from previous cluster surveys to guide sample size calculation in emergency settings. Disasters.

[B36] (2006). The World Health Survey: Sampling Guidelines for Participating Countries.

[B37] Woodruff BA (2006). Review of Survey Methodology. SMART.

[B38] (2005). World Food Programme: A Manual: Measuring and Interpreting Malnutrition and Mortality. WFP & CDC.

[B39] (2006). Standardised Monitoring and Assessment of Relief and Transitions (SMART): Measuring Mortality, Nutritional Status, and Food Security in Crisis Situations: SMART Methodology. SMART.

[B40] Myatt M, Taylor A (2006). A method for estimating mortality rates in humanitarian emergencies using previous birth history: Annex 4. FANTA project.

[B41] Becker SR, Thornton JN, Holder W (1993). Infant and child mortality estimates in two counties of Liberia: 1984. Int J Epidemiol.

[B42] Taylor WR, Chahnazarian A, Weinman J, Wernette M, Roy J, Pebley AR, Bele O, Ma-Disu M (1993). Mortality and use of health services surveys in rural Zaire. Int J Epidemiol.

[B43] Checchi F, Roberts L (2005). Interpreting and using mortality data in humanitarian emergencies: a primer for non-epidemiologists. HPN Network Paper 52, Overseas Development Institute.

[B44] Legros D, Paquet C, Nabeth P, Reed HE, Keely, CB (2001). The evolution of mortality among Rwandan refugees in Zaire between 1994 and 1997. Forced Migration and Mortality: Roundtable on the Demography of Forced Migration.

[B45] Checchi F, Elder G, Schafer M, Drouhin E, Legros D (2003). Consequences of armed conflict for an ethnic Karen population. Lancet.

[B46] Ahmad OB, Boschi-Pinto C, Lopez AD, Murray CJL, Lozano R, Inoue M (2006). Age Standardization of Rates: A New WHO Standard.

[B47] Chandramohan D, Maude GH, Rodrigues LC, Hayes RJ (1998). Verbal autopsies for adult deaths: their development and validation in a multicentre study. Trop Med Int Health.

[B48] Chandramohan D, Soleman N, Shibuya K, Porter J (2005). Ethical issues in the application of verbal autopsies in mortality surveillance systems. Trop Med Int Health.

[B49] INDEPTH Network (2006). An international network of field sites with continuous demographic evaluation of populations and their health in developing countries. INDEPTH Network.

[B50] Soleman N, Chandramohan D, Shibuya K (2006). Verbal autopsy: current practices and challenges. Bull World Health Organ.

[B51] Soleman N, Chandramohan D, Shibuya K (2005). WHO technical consultation on verbal autopsy tools. Final report: Review of the literature and currently-used verbal autopsy tools.

[B52] (2005). World Health Organization: Mortality survey among internally displaced persons and other affected populations in greater Darfur, Sudan. WHO/EPIET.

[B53] (2005). World Health Organization: Health and mortality survey among internally displaced persons in Gulu, Kitgum and Pader Districts, northern Uganda. The Republic of Uganda Ministry of Health, WHO, UNICEF, UNFPA, ICR.

[B54] Myatt M, Feleke T, Sadler K, Collins S (2005). A field trial of a survey method for estimating the coverage of selective feeding programmes. Bull World Health Organ.

